# Health Care Staff–Reported Workplace Violence in Patient Safety Event Reports

**DOI:** 10.1001/jamanetworkopen.2025.44642

**Published:** 2025-11-20

**Authors:** Azade Tabaie, Sonita S. Bennett, Alberta K. Tran, Raj M. Ratwani, Mark Marino, Josanne Revoir, Kate M. Kellogg, Laura Lee, Allan Fong

**Affiliations:** 1 Center for Biostatistics, Informatics, and Data Science, MedStar Health Research Institute, MedStar Health, Columbia, Maryland; 2 Department of Emergency Medicine, Georgetown University Medical Center, Washington, DC; 3 MedStar Health National Center for Human Factors in Healthcare, MedStar Health, Washington, DC; 4 MedStar Institute for Quality and Safety, MedStar Health, Columbia, Maryland; 5 Workplace Violence Prevention Programs, MedStar Health, Washington, DC

## Abstract

**Importance:**

Workplace violence (WPV) against health care staff is an important problem in the US and worldwide.

**Objective:**

To develop a WPV classification approach and investigate the characteristics of the WPV incidents committed against health care staff documented in patient safety event (PSE) reports.

**Design, Setting, and Participants:**

This cross-sectional study analyzed 975 self-reported PSEs recorded by health care staff from March 1 to September 20, 2023, from a multihospital health care system in the mid-Atlantic region of the US.

**Main Outcomes and Measures:**

A subset of PSE reports that potentially pertained to WPV incidents were identified through structured information of the reports. When available, the following information was captured from each report: type of WPV incident and harm, reported perpetrators and those exposed to WPV, precipitating factors to the WPV incident, reporter’s job function, whether security officers or law enforcement were contacted, and facility type.

**Results:**

A total of 15 426 PSEs were recorded. Of those, 975 reports (6.3%) were selected; for 300 (30.8%) of these reports, the free-text description of incidents was reviewed by 2 independent investigators, interrater reliability (IRR) was calculated, and the WPV classification was developed. Two investigators then independently classified the remaining 675 reports. A median IRR of 84% (IQR, 68%-99%) was achieved. Eight hundred thirty-one reports (85.2%) were related to WPV and 144 (14.8%) did not contain WPV narratives. The 831 WPV reports were further analyzed, and additional information about the WPV incidents were identified: patient- or visitor-on-staff violence (673 [76.7%]), verbal harm (331 [39.8%]), patients as perpetrators (581 [69.9%]), nurses exposed to WPV (277 [33.3%]), agitation (193 [23.2%]) and aggression (179 [21.5%]) as the leading precipitating factors, nurses reporting incidents (533 [64.1%]), contact of security officers (391 [47.1%]) and law enforcement (70 [8.4%]), and incidents occurring in hospitals (767 [92.3%]).

**Conclusions and Relevance:**

In this cross-sectional study of 15 426 PSE reports, 831 WPV incidents were identified, most involving patient- or visitor-on-staff verbal harm, with nurses frequently exposed. Agitation and aggression were the leading precipitating factors. The insights from PSE reports can inform the development of targeted WPV intervention and prevention plans, ultimately enhancing the safety of frontline staff.

## Key Points

**Question:**

What insights can patient safety event (PSE) reports provide regarding the characteristics of health care workplace violence (WPV) incidents?

**Findings:**

In this cross-sectional study of 15 426 PSE reports, 831 WPV incidents were identified. Most incidents involved patient- or visitor-on-staff violence, primarily verbal harm, with nurses frequently affected; agitation and aggression were leading precipitating factors.

**Meaning:**

These findings suggest that PSE reports offer an untapped rich source of information associated with WPV incidents that can guide targeted WPV intervention and prevention plans, ultimately enhancing the safety of frontline health care staff.

## Teaser

This cross-sectional study assesses the utility of patient safety reports as a source of information related to workplace violence against health care staff.

## Introduction

Workplace violence (WPV) is a serious problem, particularly for health care workers in the US and worldwide.^1,2^ Health care workers are at higher risk of WPV and commonly work directly with people who may have a history of violence or mental health conditions and/or who may be directly under the influence of drugs and require immediate clinical care. WPV against medical staff is mainly perpetrated by patients and patients’ family or friends.^3^

Studies have been conducted to measure WPV incidents in a randomly selected group of health care staff through surveys, questionnaires, focus groups, and interviews.^4,5,6^ Despite their importance, there is a high cost and effort associated with the implementation of organization-wide surveys, questionnaires, and interviews.^7^ Furthermore, several calls have been made by researchers and leaders to simplify incident reporting systems and thereby reduce the burden of reporting and enable data sharing across organizations to assist in the planning and evaluation of prevention strategies.^8^ Consistency and accuracy in WPV reporting practices within health care facilities has been a long-established problem for researchers, with differences in reporting expectations and efforts by organization.^8,9,10,11^

On the other hand, data recorded by staff through existing mechanisms, such as through patient safety event (PSE) reports, offer regular opportunities for staff to provide narratives of WPV, among other safety issues, and offer potential untapped, broad, system-level opportunities for improvement.^12^ PSE reports have been analyzed to better understand safety issues, including medication errors, health information technology, and intensive care unit operations.^13,14,15,16^

Self-reported PSE reports include combinations of structured and unstructured data that vary between organizations. Information representing a WPV incident can be a part of the structured elements and/or narrated in the free-text fields. Staff might not select (or have the option to select) the appropriate predefined checkbox options to flag and categorize a PSE as a WPV event; consequently, part of relevant information about WPV and other events may only occur in the free-text narratives of a PSE report. Therefore, a data-driven approach to identify WPV incidents across a health care system and analyze the reported characteristics in a structured and free-text format may overcome current limitations. Studying and creating safety surveillance systems around the characteristics of reported WPV incidents and the precipitating factors can inform ongoing WPV intervention and prevention efforts.

Without an established WPV label, staff-entered PSE reports may lack key information or alignment with standard WPV definitions, making it difficult to ascertain whether they can be appropriately used in safety surveillance systems. Therefore, creating a WPV label through manual review is a required step to building out WPV classification approaches. Creating such a label paves the way for further in-depth analysis of WPV incident characteristics and mitigating interventions and prevention efforts. In this study, we developed a qualitative WPV classification approach to guide manual review of PSE reports, which complements the information recorded in the structured fields of the reports and helps health care systems identify WPV incidents in reporting systems. Incorporating the results from the WPV classification, we further analyzed the characteristics of the reported WPV incidents identified with our classification approach.

## Methods

**Figure 1.**
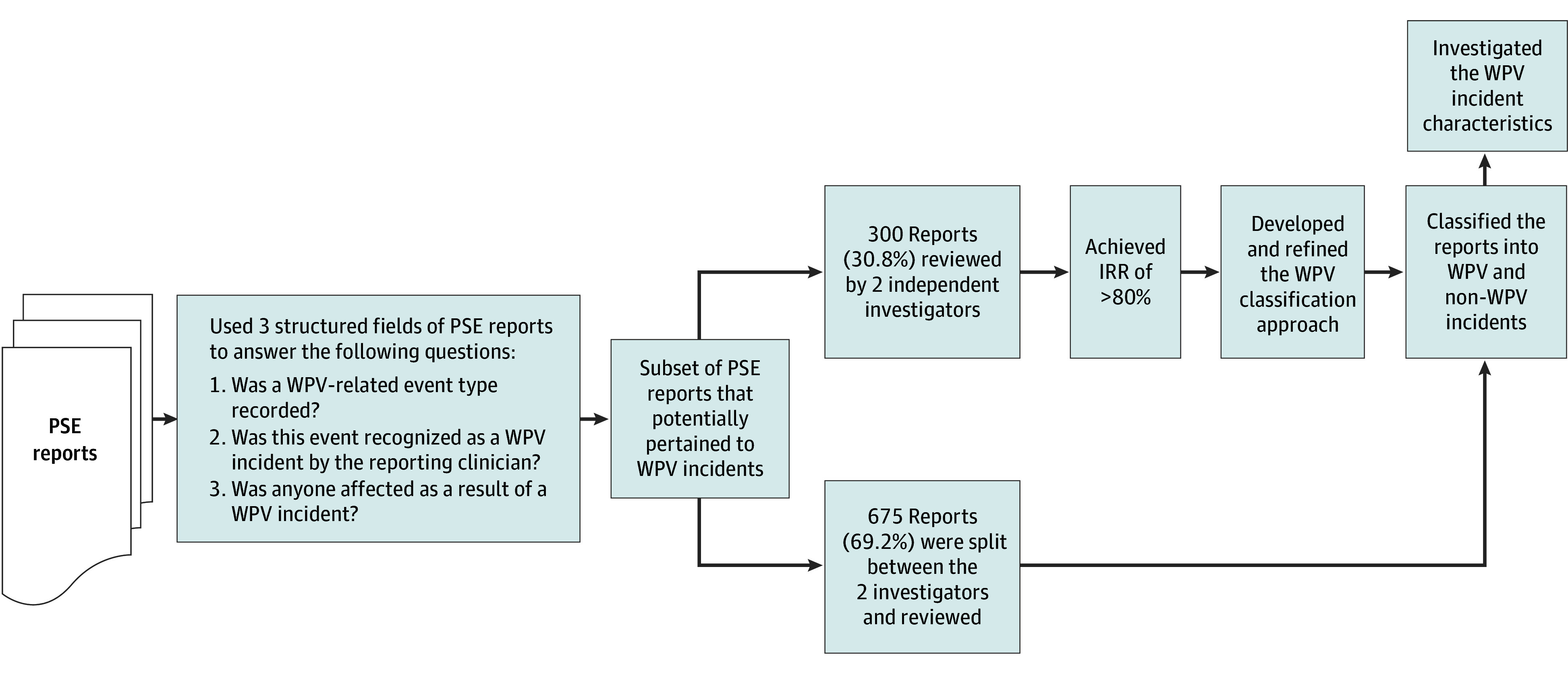
Patient Safety Event (PSE) Review Diagram IRR indicates interrater reliability; WPV, workplace violence.

This cross-sectional study was approved by the Institutional Review Board of MedStar Health, which granted a waiver of informed consent owing to the use of deidentified data from reports. Figure 1 presents a summary of our methods. We followed the Strengthening the Reporting of Observational Studies in Epidemiology (STROBE) reporting guideline.

### Setting

We evaluated PSE reports from a 10-hospital health care system in the mid-Atlantic region of the US, with 20 diversified health care entities and more than 300 outpatient service locations, including urgent care, emergency care, and primary and specialist care settings. With more than 4 million outpatient visits, 200 000 inpatient admissions, and 300 000 home visits annually, this health care system serves a racially, ethnically, and socioeconomically diverse patient population residing in urban, rural, and suburban settings. The WPV Prevention Program in the health care system has established WPV prevention staff training, incident reporting protocols, and dedicated workplace safety committees. These programs are intended to support staff safety and facilitate reporting of WPV incidents.

We focused our analysis on PSE reports in a 6-month period, recorded from March 1 to September 20, 2023. PSE reports with missing free-text incident description were dropped from the analysis. Given the high volume of PSEs, a 6-month period was selected to ensure the manual review of reports remained feasible. This 6-month time frame was chosen because reporting patterns within the study health care system have remained relatively stable across these months, making the sample reasonably representative of WPV incidents while allowing for in-depth manual classification.

When submitting a PSE report, reporters are prompted to select from a list of more than 300 event-type categories. The recorded event type categorizes a PSE according to the reason of the safety incident (eg, medication issue, patient’s fall, unauthorized weapons on the premises). However, some WPV incidents may not have been initially categorized under a WPV-related event type. To identify such cases, we also examined additional structured fields in the reports, including responses to 2 specific questions: “Was this incident recognized as a WPV incident by the reporting staff?” and “Was anyone affected as a result of a WPV incident?” This approach helped ensure that we assessed all available structured fields that may include indications of a WPV incident. The identified PSE reports underwent manual reviews.

### PSE Report Selection

Three PSE structured fields were used to identify reports that potentially described a WPV incident. We identified a WPV incident based on the Joint Commission definition: “an act or threat occurring at the workplace that can include any of the following: verbal, nonverbal, written, or physical aggression; threatening, intimidating, harassing, or humiliating words or actions; bullying; sabotage; sexual harassment; physical assaults; or other behaviors of concern involving healthcare staff.”^17^

#### Staff-Identified Event Categories Related to WPV

To identify potential WPV incidents, the study incorporated an institution-defined list of event types representing WPV-related situations. If a staff member submitted a PSE report with one of these event types—abduction, physical and/or verbal abuse, active shooter, bomb threat, disorderly conduct, domestic quarrel, hostage taking, stalking, suicide or suicide attempt, unauthorized weapons, or general workplace violence—the report was flagged as potentially WPV related.

In the PSE reporting system, *workplace violence* is a selectable event type used broadly to capture various WPV incidents. Reports may be submitted by those directly involved, witnesses, or informed parties. Notably, reports of suicide or suicide attempt often include patient self-harm narratives, but patient suicidal ideation is also a known factor associated with violence against health care staff.^18^ Therefore, such reports were reviewed for WPV content.

#### Responses to a WPV Question

Additionally, staff were asked if their report was related to WPV. An affirmative response flagged the report as potentially WPV related, even if the event type selected was not explicitly labeled as WPV.

#### Responses to a WPV Question About Harm

Finally, reporters were asked if anyone was physically harmed due to WPV. An affirmative response further supported identification of the report as WPV related.

### WPV Classification Development

The developed WPV classification method included different scenarios of WPV incidents along with deidentified examples from the free-text incident descriptions recorded in the PSE reports. To develop the WPV classification approach, 2 independent investigators (A.T. and S.S.B.) examined the free-text incident description for 300 of 975 (30.8%) potential WPV incidents recorded in PSE reports to determine which conditions in the Joint Commission WPV definition were captured. If an incident description included narratives related to any of the WPV categories, the PSE report was classified as a WPV incident and included in the final analytic sample.

The WPV classification approach was refined as both reviewers encountered potential new WPV scenarios or disagreed on classifying specific scenarios as WPV. The insight from subject matter experts (A.K.T., M.M., and J.R.) was incorporated in resolving the disagreements and refining the classification approach.

### Calculating Interrater Reliability

We examined 300 of 975 identified PSE reports, calculating the Cohen κ value and interrater reliability (IRR) analysis. The goal was to achieve a median IRR of at least 80%.^19^ The 2 independent investigators (A.T. and S.S.B.) reviewed 3 random subsets of 100 PSE reports following the WPV classification approach. Disagreements between the 2 reviewers were discussed with our subject matter experts (A.K.T., M.M., and J.R.) and reexamined. The main disagreements were around how to classify disruptive behavior and disorderly acts committed by patients. Per the subject matter recommendation, we decided to classify PSEs including disruptive behavior of patients as WPV, since such behavior could represent “behaviors of concern” in our WPV definition. For disorderly acts of patients, we looked at other clues in reports, as the term *disorderly patient* by itself cannot constitute a WPV incident (eg, an intoxicated patient who cannot follow orders may be considered disorderly while not posing a threat to staff). Finally, the median IRR of 84% (IQR, 68%-99%) was achieved.

### Coding WPV Characteristics Within PSE Reports

In developing the WPV classification approach, we focused on extracting information from WPV incidents that could inform WPV interventions and prevention plans. When available, we examined the free-text incident description of WPV incidents to identify the type of WPV incident, type of harm, staff reported as exposed to WPV, and perpetrator. Table 1 outlines different categories, definitions, and examples associated with each characteristic.

**Table 1.** Characteristics of WPV Incidents Reported in the Free-Text Incident Description

Characteristic	Definition	Example
**Type of WPV incident**		
Patient- or visitor-on-staff	Violence from patients, patients’ family members, or visitors toward staff	“While the phlebotomist was drawing patient’s blood, he became very combative and punched the phlebotomist in the face.”
Staff-on-staff	Horizontal violence, including abuse or assault from staff toward other staff, commonly verbal abuse and intimidation	“Dr X talked to me with an intimidating language in front of the OR staff during patient’s surgery.”
Other or unable to discern	Type of WPV incident was not clearly captured in the incident description	“A nurse’s partner shows up to the ED and threatens the nurse.”
**Type of harm reported**		
Verbal	Includes yelling, raising voice, intimidating, or using threatening language toward staff	“Patient was upset with the delay in discharge and yelled at nurses at the nurse station.”
Physical	Includes any physical harm or attempt at physical harm directed at staff (eg, kicking, throwing things, and attempting to bite)	“Patient’s son got angry and threw a chair at the nurse.”
Violence including suspected or threatened use of weapon	Includes incidents involving weapons including, but not limited to, knife, gun, firearm, brass knuckles, pepper spray, scissors, or baton with harm or threatening to harm directed to staff	“Patient had a pocketknife hidden in his room and stabbed the nurse with it.”
Other (eg, aggression, recording staff)	Aggression, agitation, being angry toward staff, stalking, and recording staff without permission or other forms of violence that could not be mapped	“Patient was intoxicated and agitated towards staff.”
Multiple forms of violence	Incidents involving >1 type of harm (eg, verbal abuse escalating to physical assault)	“The patient walked in calmly and registered. After registering and while being triaged, the patient punched the associate triaging her. The patient then enters the lobby and began to yell at the security officer.”
Not clear	No clear type of harm was recorded	“Went home early. Was not feeling safe.”
**Reported perpetrator**		
Patient	The patient receiving treatment is the perpetrator of WPV toward staff	“Patient used profanity towards staff.”
Visitor or family member	A visitor or family member is the perpetrator of WPV toward staff	“Daughter of the patient began yelling and cursing at staff, putting her hand in staff’s face with threatening behavior.”
Other (eg, health care staff)	Includes peer-on-peer or supervisor-on-staff WPV	“Doctor was unprofessional during our interaction and was very heated in a conversation. He was dismissive of our concerns that patient’s clinical condition was worsening and did not allow the primary doctor to express concerns fully stating ‘Let me talk. You’re not going to talk I’m going to talk.’”
Not clear	Not clearly identified in the incident report	“A letter was received by me that had my new address, my picture, and accusations of attempted murder.”
**Reported person exposed**		
Licensed independent clinician (eg, physician, advanced practice professional, nurse practitioner, physician assistant, resident)	Physician, advanced practice professional, nurse practitioner, physician assistant, and resident	“Patient made a threat of physical harm to the ED provider stating, ‘If I see him again the police will need to be called.’”
Nursing staff (eg, registered nurse, licensed practice nurse, nurses’ aide)	Registered nurse, licensed practice nurse, and nurses’ aide; also, if the person was referred to as a *nurse*, this category would be selected	“Patient jumped off the stretcher, with his fist punching and hitting the nurse.”
Security officer	Security officers	“Patient was brought in by police for suicidal ideations. Patient was aggressive and uncooperative on arrival and attempted to bite security officers during interactions.”
Patient sitter	Staff member assigned to observe the patient one-to-one	“Patient came into the hallway where the sitter was located and became combative and aggressive towards sitter and was refusing to go back into his room when asked to do so by nursing staff.”
Other (eg, radiology technician, registration staff)	Laboratory staff, radiology technicians, patient transporters and other staff members who could not be categorized in the above categories; if the affected staff member was referred to as *staff*, this category would be selected	“Patient’s son was agitated and yelled at the registration staff as he was unhappy with the waiting time in ED.”
Multiple primary	Multiple primary persons from different categories (eg, physician and nurse)	“Patient was refusing to leave the ambo area and using profanity at the nurses and the doctor.”
Not clear	Not clearly identified in the report	“Daughter of the patient began yelling and cursing at staff, putting her hand in staff’s face with threatening behavior.”

Abbreviations: ED, emergency department; OR, operating room; WPV, workplace violence.

The eAppendix in Supplement 1 presents the final WPV classification approach, including a list of possible WPV scenarios recorded in PSE reports along with deidentified examples of incident descriptions from WPV incidents. The eAppendix in Supplement 1 also contains a guide to identify the type of WPV incident, type of harm, reported individual exposed to WPV, and reported perpetrator along with examples of deidentified incident descriptions.

Although developed using our institution’s reporting system, the WPV classification approach is grounded in the Joint Commission definition of WPV and organized around characteristics (ie, type of incident, type of harm, perpetrator, and exposed individuals) that are universally applicable. As such, the framework can be adapted to other PSE systems with similar structured and narrative data fields. We documented each step of the classification process, including the coding definitions, examples, and adjudication criteria (eAppendix in Supplement 1). The 2 reviewers (A.T. and S.S.B.) independently applied these definitions, with disagreements resolved through consensus and input from the subject matter experts (A.K.T., M.M., and J.R.). This transparent approach supports reproducibility and facilitates application in other settings.

### Statistical Analysis

IRR was assessed for the subset of 300 PSE reports independently reviewed by both reviewers. The agreement was quantified using the Cohen κ coefficient, which measures concordance beyond chance. A median IRR of at least 80% was targeted to indicate acceptable reliability. Discrepancies were discussed and resolved with input from the subject matter experts. Both independent reviewers examined the remaining 675 (69.2%) of 975 PSE reports using the final WPV classification approach. Other characteristics of WPV incidents sourced from the structured fields—including facility type, whether security or law enforcement was contacted, the WPV reporter’s job function, and the precipitating factors of the incident—were analyzed. Descriptive analyses were conducted for all the reviewed WPV incidents. All analyses were performed using Python, version 3.8 (Python Software Foundation) with Pandas, version 2.0.3, and NumPy, version 1.24.4, packages.

## Results

**Figure 2.**
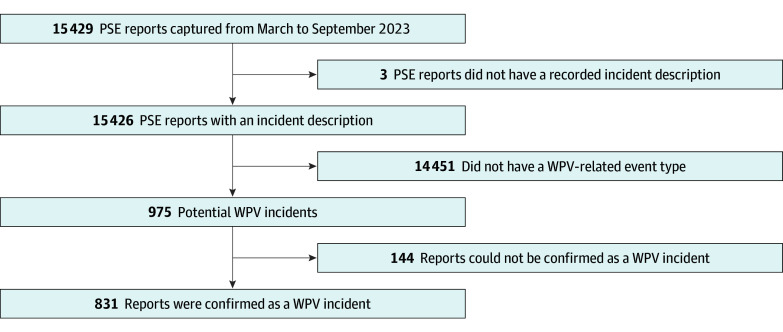
Study Inclusion and Exclusion Diagram PSE indicates patient safety event report; WPV, workplace violence.

In total, 15 429 PSE reports were captured in our safety incident reporting system from March 1 to September 20, 2023. Removing reports with missing incident narratives led to 15 426 PSEs (Figure 2).

### Classification of Potential WPV Reports

We identified 975 potential WPV incidents. Of these potential incidents, 947 (97.1%) were found to have had a WPV-related event type based on our coding definition. Only 142 PSE reports (14.6%) were labeled as a WPV incident by the reporting staff. We found that 321 PSE reports (32.9%) described an incident where health care workers were injured because of a WPV incident. Some reports were detected through more than 1 structured field.

Most PSE reports embedded a WPV incident narrative. In total, 831 (85.2%) of the 975 potential WPV incidents in PSE reports had narratives that qualified under at least 1 of the WPV categories listed in the Joint Commissions’ definition of WPV. All 831 WPV incidents had a WPV-related event type based on our coding definition. Physical and/or verbal abuse and disorderly conduct were the leading WPV-related event types observed. Further, 115 (13.8%) of 831 PSEs were labeled as a WPV incident by the reporting staff. Two hundred and ninety-four (35.4%) of 831 reports included an incident where health care workers were injured because of a WPV incident.

### Characteristics of WPV Reports

Table 2 presents the frequency of each characteristic category. All 4 characteristics were recorded in the free-text incident descriptions for 809 (97.4%) of 831 WPV incidents. Further, 673 (76.7%) of 831 WPV reports represented patient- or visitor-on-staff incidents. Staff-on-staff WPV accounted for 190 WPV reports (22.9%).

**Table 2.** Characteristics of WPV Incidents Reported in the Free-Text Incident Description

Characteristic	No (%) (n = 831)
Type of WPV incident	
Patient- or visitor-on-staff	637 (76.7)
Staff-on-staff	190 (22.9)
Unable to discern	4 (0.5)
Type of harm reported	
Verbal	331 (39.8)
Multiple forms of violence	330 (39.7)
Physical	123 (14.8)
Other form (eg, aggression, recording staff)	28 (3.4)
Violence including suspected or threatened use of weapon	18 (2.2)
Not clear	1 (0.1)
Reported perpetrator	
Patient	581 (69.9)
Visitor or family member	55 (6.6)
Other (eg, staff)	190 (22.9)
Not clear	5 (0.6)
Reported person exposed	
Nurse	277 (33.3)
Multiple primary persons	44 (5.3)
Licensed independent clinician	27 (3.2)
Security officer	25 (3.0)
Patient sitter	23 (2.8)
Other (eg, radiology technician, registration staff)	377 (45.4)
Not clear	58 (7.0)

Abbreviation: WPV, workplace violence.

Verbal violence was the leading form of harm identified in WPV reports, accounting for 331 incidents (39.8%). Multiple forms of violence were the second most common forms of harm from WPV incidents, which were inferred from 330 WPV reports (39.7%). Physical harm (123 [14.8%]), other forms of violence such as aggression and recording staff without consent (28 [3.4%]), and violence including suspected or threatened use of weapons (18 [2.2%]) were other forms of harm identified.

Patients were most frequently reported as perpetrators of WPV incidents (581 [69.9%]). Furthermore, 55 WPV incidents (6.6%) were perpetrated by visitors or family members. Staff-on-staff WPV scenarios accounted for 190 WPV reports (22.9%).

Looking at the reported individuals exposed to WPV, the most prominent category was other, with 377 WPV reports (45.4%). This category includes all WPV reports in which the exposed individual was referred to as staff. Also, individuals reported as exposed to WPV were nurses in 277 reports (33.3%), multiple primary individuals in 44 (5.3%), licensed independent health care professionals in 27 (3.2%), security officers in 25 (3.0%), and patient sitters in 23 (2.8%).

Table 3 presents the structured data recorded in WPV-related PSE reports. Most of these incidents occurred in hospitals (767 [92.3%]), with nurses being the primary reporters (533 [64.1%]). Security officers and law enforcement were contacted in 391 (47.1%) and 70 (8.4%) cases, respectively. The leading precipitating factors included agitation (193 [23.2%]), aggression (179 [21.5%]), and behavioral health diagnoses (79 [9.5%]). Notably, precipitating factors were not recorded for half of the reported incidents.

**Table 3.** Characteristics of WPV Incidents Reported in Structured Fields of the PSE Reports

Characteristic	No (%) (n = 831)
Facility type	
Hospital	767 (92.3)
Urgent care	24 (2.9)
Primary and ambulatory care	19 (2.3)
Physical therapy or rehabilitation medicine	9 (1.1)
Other (eg, specialist office, infusion center, radiology)	12 (1.4)
WPV reporter’s job function	
Registered nurse or licensed practice nurse	533 (64.1)
Patient care (excluding registered nurse or licensed practice nurse)	87 (10.5)
Physician	17 (2.0)
Imaging	14 (1.7)
Rehabilitation	11 (1.3)
Pharmacy	10 (1.2)
General staff (eg, clinical laboratory, finance, respiratory, midlevel practitioner)	82 (9.9)
Was security contacted?	
Yes	391 (47.1)
No	201 (24.2)
Unknown or not recorded	239 (28.8)
Was law enforcement contacted?	
No	488 (58.7)
Yes	70 (8.4)
Unknown or not recorded	273 (32.9)
Precipitating factor^a^	
Agitation	193 (23.2)
Aggression	179 (21.5)
Behavioral health diagnosis	79 (9.5)
Cognitive impairment	43 (5.2)
Confusion or disorientation	36 (4.3)
Substance use or intoxication	34 (4.1)
Psychiatric crisis	22 (2.6)
Delirium	16 (1.9)
Pain	12 (1.4)
Other (eg, perpetrator unhappy with search, high emotional environment surrounding death of a loved one, disagreement between staff)	47 (5.7)
Unknown or not recorded	414 (49.8)

Abbreviations: PSE, patient safety event; WPV, workplace violence.

^a^ Multiple precipitating factors may have been recorded for 1 WPV incident in a PSE report.

## Discussion

Our cross-sectional study aimed to identify WPV incidents and analyze WPV characteristics in existing PSE reporting system data. By examining the potential for free-text descriptions to be classified using established WPV definitions, our analysis offers valuable insights into identifying WPV types, precipitating factors, and law enforcement involvement. Agitation and aggression emerged as primary triggers, emphasizing the need for proactive interventions to reduce violent behavior toward staff.^20^ Additionally, behavioral health diagnoses contributed to approximately 10% of reported WPV incidents. One effective strategy used across health care settings is the implementation of assessment and screening tools for mental health conditions, such as safe assessment rooms and the Broset Violence Checklist, which evaluates violence risk through a 6-item questionnaire.^21,22^

Verbal violence was the most frequently reported form of WPV, accounting for 39.8% of all WPV reports. Additionally, verbal violence could also be captured within the category of multiple forms of violence, which constituted 39.7% of reports. These findings align with those of other studies based on surveys and interviews, where the incidence of verbal violence ranged from 66.2% to 95.1%.^5,23,24,25,26^ On the other hand, approximately one-quarter of WPV incidents contained narratives of staff-on-staff violence. This type of violence is presented as verbal abuse and intimidation. Similar to other studies, our results showed that nurses were commonly exposed to WPV, and the perpetrators could be independent health care professionals, nursing supervisors, or peers from the same nursing level.^27^

Prior studies^5,24^ have reported that physical violence accounts for 4.9% to 83.3% of WPV incidents. In our study, physical violence was recorded as a distinct category in 14.8% of reports or captured within the category of multiple forms of violence, which constituted 39.7% of incidents.^5,23,24,25,26^ Compared with verbal violence, physical violence and sexual harassment were reported less frequently. However, underreporting due to stigma or embarrassment may contribute to their lower observed frequency. PSE systems are well-established safety mechanisms that are often voluntary, and in some health care systems, including ours, can be submitted anonymously. Encouraging staff to report WPV through PSE reports may help mitigate underreporting found in surveys or other mechanisms. Similarly, optimizing the use of existing safety data collection and analysis efforts presents an untapped opportunity to capture WPV incidents that may not result in direct harm or are otherwise not reported through conventional means.

Patient- or visitor-on-staff violence was the most commonly reported type of WPV incidents, and patients were the primary reported perpetrators. Previous research^23^ also demonstrated that more than half of the WPV incidents were inflicted by patients and their family members toward staff. Our analysis also revealed that 69.9% of WPV incidents were instigated by patients.

According to our findings, patient sitters are a group of staff affected by WPV incidents. WPV against patient sitters has been called a silent epidemic. Patient sitters provide one-to-one care to patients with particular health conditions (eg, delirium or dementia). As a result, patient sitters may expect verbal or physical violence committed by patients who cannot control their behavior because of disease states. In such cases, patient sitters are unlikely to report the WPV incident and ask for support.^28^

Our findings indicate that security officers were frequently involved in WPV incidents, responding to nearly half of the cases. In certain situations, particularly in nonhospital settings where in-house security may be unavailable, staff assessments led to police involvement when additional support was needed. Our analysis showed that security officers are also called when staff are concerned about their safety. In health care settings, security officers deal with different sorts of incidents ranging from fire alarm activations to de-escalating a violent situation between staff and patients or family members.^29^ Calling a security officer happens when a situation is out of control, for instance, when a patient who was diagnosed with severe substance abuse shows withdrawal symptoms.^25^ For instance, security presence and assistance were requested when restraints were being applied following a physician’s order or when medication was administered for a patient with a history of violence toward staff. Intervening in such situations puts security officers at higher risk of being exposed to WPV.

The information in PSE reports provides insights into some of the resolution strategies used by staff in response to WPV incidents. Although we did not purposefully examine resolution strategies in this study, future studies could review PSE reports to assess and characterize the resolution strategies reported by staff, reviewing the deployment of and impacts of, for example, patient medication, restraint, or verbal de-escalation on reported incidents.

### Strengths and Limitations

As an early pilot study, our work provides a road map for building more sophisticated WPV surveillance methods. The manual classification approach demonstrated feasibility and reliability, establishing a foundation for future research that could apply natural language processing and machine learning techniques to automate WPV detection in PSE reports. This trajectory enhances methodologic rigor and scalability while maintaining alignment with established WPV definitions.

However, this study has some limitations. We relied on self-reported data that exist as part of our safety surveillance, but these data only reflect the tip of a much bigger iceberg. Integrating additional sources of reported WPV may improve the accuracy and comprehensiveness of the analysis and help overcome barriers to underreporting of these incidents. Future studies should consider the inclusion of other WPV-related data recorded across health care systems (eg, occupational health data [Occupational Health and Safety Administration form 300]) to provide more comprehensive insights into the characteristics of WPV incidents reported by staff and better understand concordance and gaps between reporting systems.

Although our classification approach was developed within a single health care system using a commercial reporting platform with locally customized, WPV event type categories, it is anchored to the Joint Commission definition of WPV and broad categories of harm, perpetrator, and exposure, making it adaptable to other PSE systems. Future validation in external settings is needed to confirm generalizability.

Our analysis was limited to a 6-month period, selected for feasibility of manual review and classification. Although reporting trends within the study health care system have been relatively stable across months, the findings may not capture potential seasonal or temporal variation in WPV reporting.

WPV events were identified using structured fields within PSE reports and subsequently confirmed or ruled out through manual reviews. While event type is a commonly recorded element in PSE reporting systems, the other 2 data points—whether the reporting health care worker recognized the event as WPV and whether individuals were affected as a result—may not be routinely captured in PSEs from other health care systems, potentially limiting generalizability. However, the inclusion of event type in PSE reports is standard across many health care systems, which may support the broader applicability of findings based on this data element.

In lieu of a gold-standard WPV label for PSE reports, we developed a WPV classification method that was labor intensive and time consuming to implement. However, our WPV classification model was an effort to generate preliminary data that may be incorporated with large language models and artificial intelligence techniques to develop automated WPV classification models.

In this study, we consolidated patient-on-staff and visitor-on-staff violence into a single category to align with the standard definition of type II WPV. However, these 2 groups may involve different triggers and require distinct intervention strategies. Future work should consider disaggregating these categories to enable more targeted analysis and intervention planning.

## Conclusions

This cross-sectional study found PSEs are routinely recorded in health care systems and serve as a rich source of information on safety incidents, including those related to WPV. Analyzing WPV incidents documented in PSE reports offers valuable insights into their characteristics and precipitating factors. This analysis can inform the development of more effective WPV intervention and prevention plans, ultimately enhancing the safety of frontline health care staff.

## Notes

Supplement 1. **eAppendix.** Workplace Violence Classification Approach and Incident Characteristics

Supplement 2.
Data Sharing Statement
