# Supplementary material for: Health Care Staff–Reported Workplace Violence in Patient Safety Event Reports
Source: JAMA Netw Open. 2025 Nov 20;8(11):e2544642. doi: 10.1001/jamanetworkopen.2025.44642 (PMC12635879; doi:10.1001/jamanetworkopen.2025.44642)
Supplement: Supplement 1. — eAppendix. Workplace Violence Classification Approach and Incident Characteristics [file jamanetwopen-e2544642-s001.pdf]

Supplementary Online Content

Tabaie A, Bennett SS, Tran AK, et al. Health care staff–reported workplace violence in patient safety event reports. *JAMA Netw Open.* 2025;8(11):e2544642. doi:10.1001/jamanetworkopen.2025.44642

eAppendix. Workplace Violence Classification Approach and Incident Characteristics

This supplementary material has been provided by the authors to give readers additional information about their work.

© 2025 Tabaie A et al. *JAMA Network Open*.

eAppendix. Workplace Violence Classification Approach and Incident Characteristics

Workplace Violence Classification Approach

Workplace violence (WPV) definition according to The Joint Commission: "An act or threat occurring at the workplace that can include any of the following: verbal, nonverbal, written, or physical aggression; threatening, intimidating, harassing, or humiliating words or actions; bullying; sabotage; sexual harassment; physical assaults; or other behaviors of concern involving staff."

In this definition of WPV, any violence that was not directed to healthcare staff was not counted as WPV incident.

The focus of this study is on WPV incidents in hospital settings only (inpatient and ED) towards healthcare staff. Home healthcare and ambulatory care will be analyzed in future studies.

Please note: if your answer to question 1 is "Yes", then move on to the next four questions. Otherwise, questions 2 to 5 should not be answered.

Is the report explicitly relevant to WPV (single select)?

a. **Yes, the report is relevant.**
   Relevant if the event narrative described a violent act and/or direct threat of a violent act, that occurred at work and the violence was directed towards healthcare staff.
   - "Attempt to kick the nurse" is WPV incident.
   - Domestic altercations are not WPV incidents unless a type of harm was directed towards healthcare staff.
     - ❖ Example of WPV incident: "there was a domestic altercation in ED. The triage nurse tried to de-escalate the situation, but she was yelled at by the patient."
     - ❖ Example of Non-WPV incident: "there was a domestic altercation in ED. Patient family member was yelling at the patient."

   Relevant if the incident involved the use or threaten to use a weapon (e.g., gun, knife, cigarette lighter, or other weapons) on healthcare staff.
     - ❖ Example: "patient was not happy with the ED wait time. He approached the triage nurse and said "Maybe I need to come back here with a loaded gun.""
     - ❖ Example: "patient in room A attacked the nurse with a knife she had hidden in her belongings."

   Relevant if the incident involved gun, knife, or other weapons which was not used to harm healthcare staff, but the objects were found at places which were not expected (e.g., patient room, emergency department, etc.). The only place which we expect so see and confiscate weapon is security check points.
     - ❖ Example: "A loaded gun magazine with 10 rounds of ammunition was discovered in patient's room."
     - ❖ Example: "Nurse suspected that the patient in room A had a knife hidden in his belongings."

   Relevant if security officers responded to a rapid response or code BERT to de-escalate a violent/aggressive patient. It is not relevant to WPV if the report does not include

© 2025 Tabaie A et al. *JAMA Network Open*.

indications of violent acts toward healthcare staff or does not include “harm to others” as the reason for restraining a patient.

- ❖ Example of non-WPV incident: “code BERT was called to room A. The doctor and BERT team wanted assistance with the patient for meds. Patient was given medicine with no incident.”
- ❖ Example of non-WPV incident: “code BERT was called on room A. BERT team have arrived. Patient was placed in two-point restraint per doctor’s order.”
- ❖ Example of WPV incident: “code BERT was called on room A. Upon arrival, patient was combative and yelled at nurses. Patient was placed in two-point restraint per doctor’s order.”

Relevant if security was called to escort a terminated employee out and the situation required de-escalation.

- ❖ Example of non-WPV incident: “security officer presented while the terminated employee cleared his locker. Then escorted him out of the hospital.”
- ❖ Example of WPV incident: “terminated employee was upset, raised his voice, and demanded to talk to his supervisor. Security officer presented while the terminated employee cleared his locker. Then escorted him out of the hospital.”

Elopement is not considered a WPV unless there was a harm towards healthcare staff in the elopement process.

- ❖ Example of non-WPV incident: “security officers were notified of a patient elopement.”
- ❖ Example of WPV incident: “security officers were notified of a patient elopement. A nurse stated that the patient pushed her away and ran away from his room.”

Relevant if patient showed erratic behavior which was followed by restraining or medicating the patient or calling for back up. This one fits under “behavior of concern involving staff” in the workplace violence definition. Disorderly will be coded similar to erratic behavior.

- ❖ Example: “patient was place in ed rm #n. since the patient exhibited erratic behavior, he was wanded by security officer and immediately placed in four-point restraints by ed staff.”

Relevant if patient/visitor threw thing around, even if it was not targeted towards healthcare staff.

- ❖ Example: “patient got upset and threw food tray to the wall.”

Relevant if “de-escalation” was mentioned with other clues (e.g., code green). There may be no description of the incident leading to code green and de-escalation.

- ❖ Example: “security responded to the code green on telemetry in room n where we had verbally de-escalated the patient and then the code was cleared. we hung out for a while to make sure everything was ok. nothing else follows.”

© 2025 Tabaie A et al. *JAMA Network Open*.

Relevant if patient/visitor took pictures or videos from healthcare staff without authorization.

- Example: ”nursing supervisor from the emergency room approached security officer and stated that a patient at the triage waiting room was recording without authorization. the security officer came in contact with the patient and explained the hospital regulations. patient willingly complied with the staff and situation was concluded.”

Relevant if patient is depressing the panic alarm repeatedly. This is outside normal behavior and prevent the staff from providing care to other patients. This fits into “behavior of concern involving healthcare staff” in the workplace violence definition. On the same note, also relevant if a patient press fire alarm even once when there is no need as it interfere with the care providing to other patients. So, it again fits into “behavior of concern involving healthcare staff” in the workplace violence definition.

- Example: “patient in behavioral health unit was repeatedly depressing the panic alarm in her room. she was refusing to follow directions from medical staff.”

b. No, the report is not relevant.

There was no mention of harm or threat directed to healthcare staff. No need to answer the rest of the questions in the codebook.

- Example: “patient was admitted and placed in ER room. She is being detained by a Police Officer. She is under arrest for assault while she receives treatment.”
- Example: “patient was assaulted by her significant other. Medic #3 brought her to the ER for treatment.”
- Example: “no weapon was found on the visitor after wanding.”
- Example: “patient walked into the ER with a gunshot wound. He was taken to ER adult code where medical staff began treatment.”

Not relevant if patient/visitor was wanded, a weapon (e.g., gun, knife, cigarette lighter, or other weapons) was found and confiscated without an issue. Finding weapons at security check points during wanding is expected so it is not workplace violence.

- Example: “After wanding the visitor, I discovered a green cigarette lighter”.
- Example: “A loaded gun was found on visitor’s pocket. The security officer asked the visitor to leave his gun in the car then comeback to visit his patient. The visitor followed the instruction.”

Not relevant to WPV if a security officer or other healthcare staff was called to be on standby for a **potential** WPV incident and there were no WPV incident at the end (i.e., no harm to healthcare staff).

- Example: “security officers responded to telemetry to be on standby for medication for a confused patient. No assistance was needed only security presence without incident.”

© 2025 Tabaie A et al. *JAMA Network Open*.

Not relevant to WPV if the patient/visitor have history of being violent towards the healthcare staff in prior visits but nothing was mentioned about an act of violence in the current report.

- ❖ Example: “patient’s son threatened to attack the nurse with a pocketknife last time he visited his mother. So, the nurse called security officer to wand the visitor this time. Nothing was found on him.”

Not relevant if the patient/visitor was talking loudly.

- ❖ Example: “patient was not satisfied with the ED wait time and he was talking loudly and expressing his unhappiness to the triage nurse.”

Not relevant if patient showed erratic behavior (i.e., was not following orders) but there were no actions taken (no medicating, restraining the patient, or calling for back up). The possible “happy high” situation.

- ❖ Example: “associate from behavioral health unit requested that the patient, who entered the emergency department psych annex, voluntarily, be wanded, as a safety precaution due to her erratic behavior.”

Not relevant if patient was “disruptive” but there was no issue (e.g., did not throw thing).

- ❖ Example: “patient was being disruptive and wanted to leave the unit. he was advised by doctor that he wasn't able to leave. the patient was medicated and escorted back to the room without incident.”

Not relevant if patient/visitor damages hospital property but was no threat to the healthcare staff.

- ❖ Example: “officer was informed that patient hit the exit sign attached to the ceiling and cause it to lean over. the patient vandalize the sign and facility were called for repair. medical personnel spoke with patient and all clear. no injuries to staff or patient reported.”

Workplace Violence Incidents Characteristics

1. **What type of WPV is this (Type 2 = client-on-worker), or (Type 3 = worker-on-worker) (single select)?**
   a. **Client-on-worker (also known as Type 2 WPV)**
   In Type 2 violence is the most common in healthcare settings. This type will be also referred to as client-on-worker violence. In healthcare environment, client includes patients, their family members, and visitors. Research shows that this type of violence occurs most frequently in emergency and psychiatric treatment settings, waiting rooms, and geriatric settings, but is by no means limited to these.
   b. **Violence by colleagues and staff to a healthcare provider (Type 3 WPV)**
   WPV may occur between coworkers. It includes bullying, and frequently manifests as verbal and emotional abuse that is unfair, offensive, vindictive, and/or humiliating.
      - ❖ Example: “Dr. X talked to me with an intimidating language in front of the OR staff during patient’s surgery.”
   c. **Other/Unable to discern.**

© 2025 Tabaie A et al. *JAMA Network Open*.

Acts of violence with criminal intent, sometimes with or without a personal association to the healthcare provider (Type 1 and Type 4 WPV)

In some cases, the perpetrator has no legitimate relationship to the business or its employees and can be committing a crime in conjunction with the violence (robbery, shoplifting, trespassing) (Type 1)

- ❖ Example: a nurse was assaulted in the hospital parking garage.
- ❖ Example: a home health care nurse is mugged while conducting a home visit.

Other times, the violence might occur due to a personal association to the healthcare provider (Type 4). We code these reports under “other/unable to discern” category as well.

- ❖ Example: a nurse’s partner shows up to the workplace and threatens the nurse

2. What was the type of harm (single select)?

a. Physical

Any physical harm or attempt to physical harm directed at healthcare staff.

- ❖ Example: “patient attempted to bite the nurse’s hand.”
- ❖ Example: “patient kicked the nurse.”
- ❖ Example: “patient’s son got angry and threw a chair at the nurse.”

If only the word “assault” was mentioned without clarification, the type of harm will be coded as physical.

- ❖ Example: “visitor assaulted the security officer.”

b. Verbal

Yelling, raising voice, verbally abusive or threatening language directed to healthcare staff.

- ❖ Example: “patient’s son threatened to burn the place down.”
- ❖ Example: “patient was upset with the delay in discharge and yelled at nurses at the nurse station.”

c. Violence including suspected or threatened use of weapon.

Violence including weapon includes incidents involving knife, gun, firearm, brass knuckles, pepper spray, scissor, or baton with harm or threatening to harm directed to healthcare staff.

- ❖ Example: “patient had a pocketknife hidden in his room and stabbed the nurse with it.”

d. Multiple forms of violence

- ❖ Example: “Patient has been verbally abusive toward staff at start of shift yelling and cursing. Patient threw urine all over the floor including the hallway.”
- ❖ Example: “The patient walked in calmly and registered. After registering and while being triaged, the patient punched the associate triaging her. The patient then enters the lobby and began to yell at the security officer.”

e. Other (please specify)

One of the “other” forms of violence is when a patient/visitor shows aggression towards staff.

- ❖ Example: “patient was agitated towards staff.”
- ❖ Example: “patient was aggressive towards staff.”
- ❖ Example: “patient was combative.”
- ❖ Example: “patient was acting out.”
- ❖ Example: “visitor was angry.”

Other types of violence also include repetitively stealing from or stalking healthcare staff. This fits into harassing someone at work.

Other types of violence also include harassment, acting belligerent, and acting out of control.

Other types of violence also include recording or taking picture of staff. If the patient/visitor was warned about the action and they deleted the picture/video, then it is not WPV.

Relevant if staff was stalked by patient or visitors.

Relevant if a patient intentionally exposed themselves to staff. But not relevant if the patient was brought to the facility with no clothes on.

Intentional urinating or defecating is workplace violence but urinating as a result of a medical condition (e.g., seizure) is not workplace violence.

f. **Not clear (potentially not explicitly related to WPV)**

3. **Who was the perpetrator (single select)?**

This study is focused on client-on-worker (type 2) WPV. So, the perpetrator will be coded as one of the following.

a. **Patient**

b. **Visitor/family member**
   - ❖ Example: “Daughter of the patient began yelling and cursing at staff, putting her hand in staff's face with threatening behavior.”

c. **Other**
   If worker-on-worker violence was detected, then the perpetrator will be coded as “other”.
   - ❖ Example: “Dr was unprofessional during our interaction and was very heated in a conversation. He was dismissive of our concerns that patient's clinical condition was worsening and did not allow the primary doctor to express concerns fully stating "let me talk. you're not going to talk I'm going to talk".”

d. **Not clear**

4. **Who was the primary victim of the WPV incident (single-select)?**
   In a WPV incident, more than one healthcare staff might be the victim. For example, “patient kicked her sitter and pushed the nurse to elope from the room.” In this case, patient sitter and

© 2025 Tabaie A et al. *JAMA Network Open*.

nurse were both the victims. However in this data annotation, we look at the **primary** victim of a WPV incident.

a. **Licensed independent provider (e.g., physician, Advanced Practice Provider (APP), nurse practitioner, physician assistant, resident)**
b. **Nurse (e.g., registered nurse, licensed practice nurse, nurses' aid)**
   If the type of nurse was not recorded in the report, then we use this category. Also, nurses' aide may be referred to as "CNA" certified nurse assistant (?) or nurse aide.
c. **Security officer**
d. **Patient sitter**
e. **Other healthcare staff or employee (please specify)**
   When the title of the victim is clearly mentioned but it does not fit into the four categories above.
   - ❖ Example: "MRI clinical aide went to retrieve the below patient for her MRI. The MRI clinical aide greeted the patient upon entering the room, but the patient got out of her bed and was yelling. She then threw a drink at the MRI clinical aide."
f. **Multiple primary victims**
   When harm was done towards healthcare staff with different status at the same time.
   - ❖ Example: "patient was refusing to leave the ambo area and using profanity at the nurses and the doctor."
g. **Not clear**
   In some reports, there are victims of workplace violence, but their title is not clearly mentioned. Sometimes, staff are referred to as "staff" which does not clarify the victims' titles. So, they will be categorized as "not clear".
   - ❖ Example: "Daughter of the patient began yelling and cursing at staff, putting her hand in staff's face with threatening behavior."

© 2025 Tabaie A et al. *JAMA Network Open*.
