# Supplementary material for: Health Care Staff–Reported Workplace Violence in Patient Safety Event Reports
Source: JAMA Netw Open. 2025 Nov 20;8(11):e2544642. doi: 10.1001/jamanetworkopen.2025.44642 (PMC12635879; doi:10.1001/jamanetworkopen.2025.44642)
Supplement: Supplement 2. — Data Sharing Statement [file jamanetwopen-e2544642-s002.pdf]

Data Sharing Statement

Tabaie. Health Care Staff–Reported Workplace Violence in Patient Safety Event Reports.
JAMA Netw Open. Published November 20, 2025. doi:10.1001/jamanetworkopen.2025.44642

Data
Data available: No
